# Working Memory Impairment in Fibromyalgia Patients Associated with Altered Frontoparietal Memory Network

**DOI:** 10.1371/journal.pone.0037808

**Published:** 2012-06-08

**Authors:** Jeehye Seo, Seong-Ho Kim, Yang-Tae Kim, Hui-jin Song, Jae-jun Lee, Sang-Hyon Kim, Seung Woo Han, Eon Jeong Nam, Seong-Kyu Kim, Hui Joong Lee, Seung-Jae Lee, Yongmin Chang

**Affiliations:** 1 Department of Medical and Biological Engineering, Kyungpook National University, Dong-In dong, Jung-gu, Daegu, Korea; 2 Division of Rheumatology, Inje University College of Medicine, Haeundae Paik Hospital, Jwa-dong, Busan, Korea; 3 Department of Psychiatry, School of Medicine, Keimyung University, Daegu, Korea; 4 Division of Magnetic Resonance, Korea Basic Science Institute, Ochang, Cheongwon, Chungbuk, Korea; 5 Department of Internal Medicine, School of Medicine, Keimyung University, Dong-san dong, Daegu, Korea; 6 Division of Rheumatology, Department of Internal Medicine, Daegu Fatima Hospital, Shin-am dong, Daegu, Korea; 7 Department of Internal Medicine, Kyungpook National University College of Medicine, Dong-In dong, Jung-gu, Daegu, Korea; 8 Department of Internal Medicine, Arthritis and Autoimmunity Research Center, Catholic University of Daegu School of Medicine, Dae-myung dong, Daegu, Korea; 9 Department of Radiology, Kyungpook National University College of Medicine, Dong-In dong, Jung-gu, Daegu, Korea; 10 Department of Psychiatry, Kyungpook National University College of Medicine, Dong-In dong, Jung-gu, Daegu, Korea; 11 Department of Molecular Medicine, Kyungpook National University College of Medicine, Dong-In dong, Jung-gu, Daegu, Korea; University of Maryland, College Park, United States of America

## Abstract

**Background:**

Fibromyalgia (FM) is a disorder characterized by chronic widespread pain and frequently associated with other symptoms. Patients with FM commonly report cognitive complaints, including memory problem. The objective of this study was to investigate the differences in neural correlates of working memory between FM patients and healthy subjects, using functional magnetic resonance imaging (MRI).

**Methodology/Principal Findings:**

Nineteen FM patients and 22 healthy subjects performed an n-back memory task during MRI scan. Functional MRI data were analyzed using within- and between-group analysis. Both activated and deactivated brain regions during n-back task were evaluated. In addition, to investigate the possible effect of depression and anxiety, group analysis was also performed with depression and anxiety level in terms of Beck depression inventory (BDI) and Beck anxiety inventory (BAI) as a covariate. Between-group analyses, after controlling for depression and anxiety level, revealed that within the working memory network, inferior parietal cortex was strongly associated with the mild (*r* = 0.309, *P* = 0.049) and moderate (*r* = 0.331, *P* = 0.034) pain ratings. In addition, between-group comparison revealed that within the working memory network, the left DLPFC, right VLPFC, and right inferior parietal cortex were associated with the rating of depression and anxiety?

**Conclusions/Significance:**

Our results suggest that the working memory deficit found in FM patients may be attributable to differences in neural activation of the frontoparietal memory network and may result from both pain itself and depression and anxiety associated with pain.

## Introduction

Chronic widespread musculoskeletal pain and multiple tender points are characteristic of fibromyalgia (FM). A high proportion of patients with FM also experience various other symptoms such as depression, fatigue and sleep disturbances [1]. Patients with FM commonly report cognitive complaints, including memory and attention problems [2], [3]. These memory and concentration problems in FM patients were related to impairments in their ability to organize and plan ahead, express themselves, respond quickly to questions, and to drive [4]. There is mounting evidence to suggest that cognitive deficits are more prevalent in FM patients compared with controls [5], [6]. On a variety of tests of working memory, FM patients showed lower performance relative to controls [7], [8].

Recent neuroimaging studies have provided growing evidence to support the view that FM patients have various kinds of abnormalities in the frontoparietal networks [9]–[14]. Using voxel-based morphometry, previous studies demonstrated decreased gray matter volumes in the frontal [14] and parietal cortex [12] in FM patients. Single photon emission computed tomography in FM patients revealed parietal hyperperfusion and frontal hypoperfusion relative to healthy subjects [11]. Functional magnetic resonance imaging (fMRI) showed that FM patients exhibited greater activation in the frontal [9] and parietal cortex [10] compared with healthy subjects in response to nonpainful stimuli. Furthermore, Luerding et al. [13] specifically attempted to link neuropsychological deficits to brain morphology in FM patients.

To the best of our knowledge, however, no investigations have directly examined neural processing during performance of working memory in FM patients, except for a brief report [15]. Therefore, the first goal of the present study is to elucidate the differences in neural correlates of working memory between FM patients and healthy subjects, using fMRI. We employed an n-back task which has been previously employed to investigate the neural basis of working memory processes [16], [17]. We hypothesized that FM patients would show abnormal brain activity in the frontoparietal memory network relative to that in healthy subjects. Because patients with FM demonstrated higher scores in ratings of depression and anxiety, we also investigated the effect of depression and anxiety on the disruption in the frontoparietal memory network in patients with FM.

In addition, we investigate possible differences in the deactivation brain network between FM patients and control subjects during performance of the n-back memory task. It has been suggested that brain areas such as posterior cingulate cortex, lateral parietal areas and anterior cingulate cortex, which were deactivated during task, closely correlate with performance in the working memory task [18], [19]. Therefore, in addition to the activation network, it is important to investigate the deactivation network during the n-back working memory task, to elucidate the coordinated modulation of neural activity for successful memory functioning.

## Results

### 1. Demographics and Characteristics of Enrolled Subject

The general characteristics of the enrolled subjects are presented in Table 1. There was no statistically significant difference in age or education level between the two groups (*P* = 0.96 and *P* = 0.18, respectively). The mean disease duration of FMS was 39.41±43.90 months, with the FMS patients showing average tender points of 13.37±4.00, average BFI score of 6.62±2.60, and average FIQ score of 59.37±19.89. BAI and BDI scores of FMS patients were significantly different from those of the controls (*P*<0.01 of both). Healthy controls did not have any tender points. The difference in intelligent quotient (IQ) between the two groups was not statistically significant (*P* = 0.08).

**Table 1:** Demographic and clinical information for fibromyalgia and control groups.

	Fibromyalgia	Healthy controls	Statistics
	N = 19	N = 22	p-value
Demographic data			
Age (years)	38.73±7.65	38.27±8.48	0.96
Education level	12.78±2.27	13.72±2.16	0.18
Clinical data			
Tender points	13.37±4.00	–	–
FIQ	59.37±19.89	–	–
BFI	6.62±2.60	–	–
Disease duration (months)	39.41±43.90	–	–
Psychological data			
BDI	23.21±10.59	9.36±6.28	<0.01
BAI	29.79±8.45	8.91±8.55	<0.01
IQ	106.10±12.23	113.04±12.39	0.08
Pressure-pain intensity (kg/cm^2^)			
Mild	1.83±0.35	2.47±0.66	<0.01
Moderate	3.27±0.80	4.33±0.95	<0.01
Task performance			
Response time (msec)			
0-back	887.81±117.44	768.33±91.68	<0.01
2-back	1080.73±163.80	966.54±113.41	<0.05
Task accuracy (%)			
0-back	98.84±2.81	99.58±0.96	0.6
2-back	88.26±13.116	95.56±3.88	<0.05

FIQ, Fibromyalgia impact questionnaire; BFI, Brief fatigue inventory; BDI, Beck depression inventory; BAI, Beck anxiety inventory; IQ, Intelligence quotient.

### 2. Pain Threshold Measurement

FMS patients showed significantly lower pressure pain thresholds at mild and moderate pain intensity levels compared with those showed by control subjects (*P*<0.01 of both) (Table 1).

### 3. Comparison of N-back Task Performance

In terms of accuracy and response time, mean performance of n-back tasks was inferior in the FM group compared with the control group (Table 1). Differences in task accuracy and response time between the two groups were statistically significant (*P*<0.05) except for 0-back accuracy.

### 4. Activation

**Figure 1:**
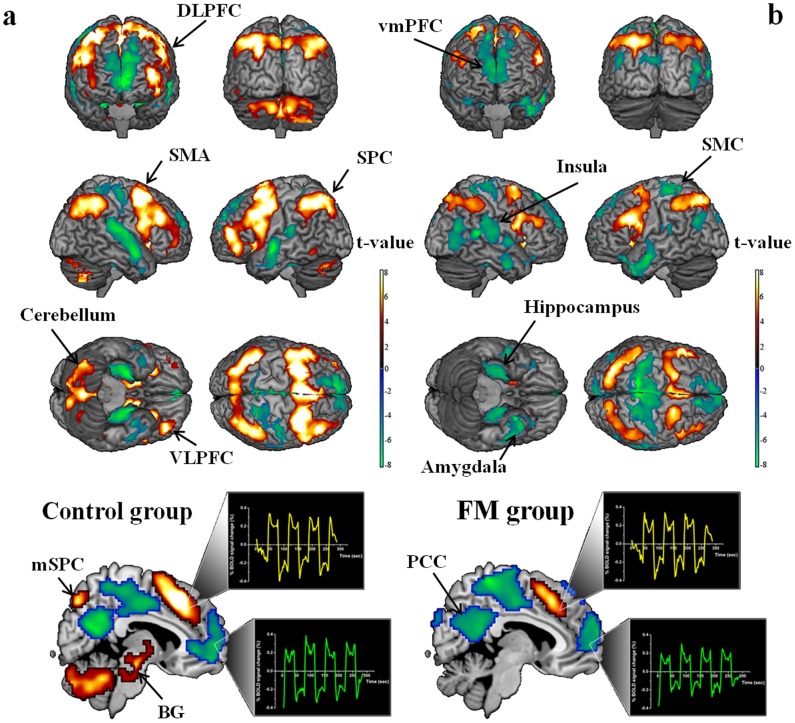
Within group analysis of n-back task. One-sample t-test group comparison in the control group (a) and in the fibromyalgia (FM) group (b). Both the task related activation regions (red to yellow) and the task related deactivation regions (blue to green) were represented in 3D brain template. The representative time course of blood oxygen level dependent (BOLD) signal at the dorsal medial prefrontal cortex (mPFC) demonstrated a positive signal change (yellow) during n-back task while the time course of BOLD signal at the anterior cingulate cortex (ACC) showed a negative signal change (green) during n-back task. The SPM{t}s were thresholded at *P*<0.01, false discovery rate (FDR)-corrected for multiple comparisons across the whole brain for activation and deactivation. DLPFC: dorsolateral prefrontal cortex, SMA: supplemental motor area, SPC: superior parietal cortex, VLPFC: ventrolateral prefrontal cortex, BG: basal ganglia, vmPFC: ventral medial prefrontal cortex, PCC: posterior cingulate cortex.

**Figure 2:**
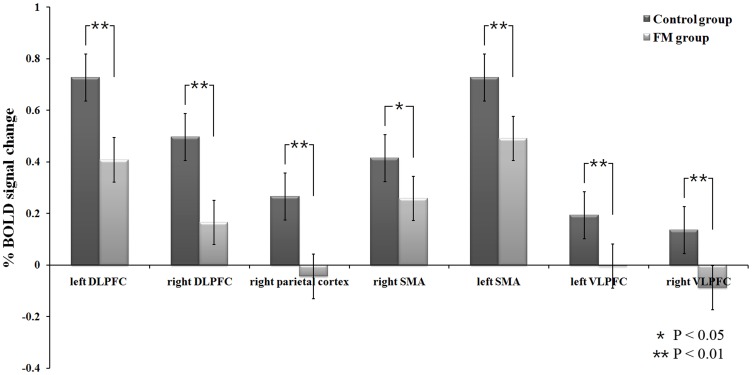
BOLD signal changes for 2-back task. Mean percentage changes of BOLD signal for 2-back memory task in the control group (blue) and in the fibromyalgia (FM) group (red). While both groups showed an activation of working memory network, the control group has stronger BOLD activity than that of the FM group at the activated brain regions. At the right parietal cortex and the right VLPFC, the FM patients even showed negative activity. *p<0.05, **p<0.01, significant difference of two groups.

**Figure 3:**
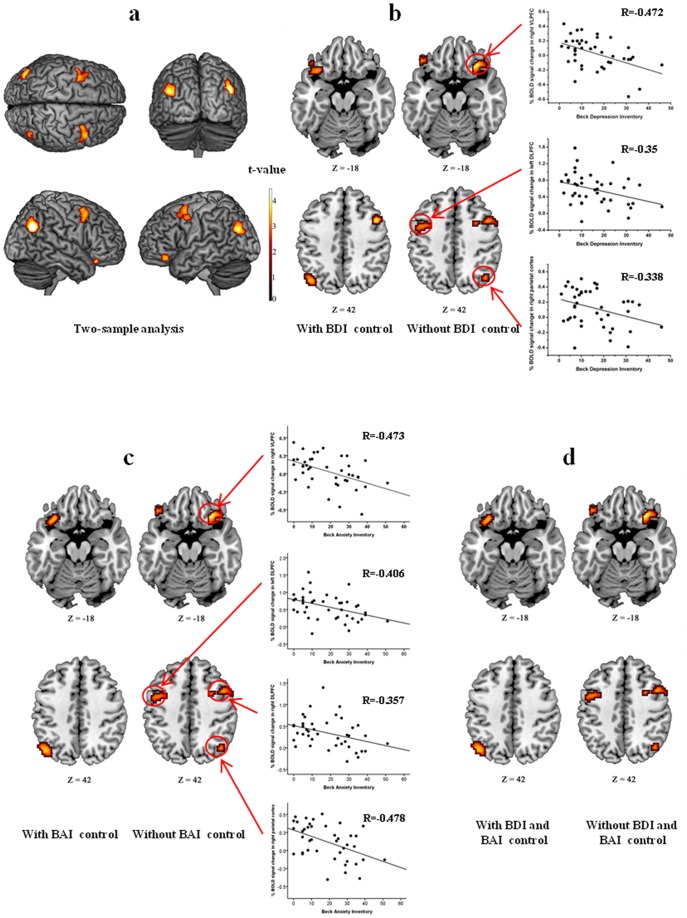
Between group analysis of n-back task. (a) Two-sample between group analysis exhibited significantly higher activation in the control group than the FM group in the VLPFC, the thalamus, middle temporal cortex, inferior parietal cortex (*P*<0.05, FDR-corrected for multiple comparisons at the voxel level). (b) Between group analyses before and after controlling depression (BDI) demonstrated that right VLPFC, the left DLPFC, and right inferior parietal cortex are strongly associated with depression. (c) Between group analysis before and after controlling anxiety (BAI) demonstrated that right VLPFC, right DLPFC, left DLPFC, and right inferior parietal cortex are strongly associated with anxiety. The correlation analysis of the BOLD activities in these brain regions with BDI scores further demonstrated the association of these regions with depression. (d) Between group analysis before and after controlling both depression (BDI) and anxiety (BAI).

Within-group analyses, which were thresholded at *P*<0.01, false discovery rate (FDR)-corrected for multiple comparisons across the whole brain, showed activity in the network of the frontal and parietal cortical areas in both the FM and control groups for the 2-back working memory task (Fig. 1). The network included activation in the ventrolateral prefrontal cortex (VLPFC), inferior temporal cortex (ITC), dorsolateral prefrontal cortex (DLPFC), dorsal medial prefrontal cortex (mPFC), and the inferior and superior parietal cortices (Table 2). Mean percentage changes in BOLD fMRI signal of each group in the activated brain regions (Fig. 2) show that for a given 2-back task, the control group had stronger BOLD activity than did the FM group. Direct comparison between the groups showed that during the memory task, the control group showed higher activation than the FM group in the left DLPFC and inferior parietal cortex. (Fig. 3 and Table 3). No region showed significantly higher activation in the FM group than in the control group. Among FM patients, two-sample group analyses, which were thresholded at *P*<0.01 uncorrected, showed that there were no differences in activation patterns between patients without antidepressants and patients with antidepressants and also between all FM patients and patients with antidepressants.

**Table 2:** 2-back minus 0-back activation one sample t-test (FDR corrected for multiple comparison, *P*<0.01 and minimum cluster size of 64).

		Fibromyalgia					Healthy Controls				
	Side	Cluster size	x	y	z	Peak T	Cluster size	x	y	z	Peak T
Dorsolateral prefrontal cortex	L	982	−48	15	24	9.42	1535	−42	9	30	16.13
	R	751	51	9	21	9.58	1549	30	9	57	15.10
Ventrolateral prefrontal cortex	L	167	−33	25	−6	7.15	272	−33	24	−6	9.91
	R	112	33	26	−8	7.00	123	33	26	−6	9.20
Dorsal medial prefrontal cortex	L	58	−6	20	47	4.40	150	−6	24	45	10.91
	R	36	6	27	42	5.45	114	9	27	45	11.82
Supplementary motor areas	L	193	−6	3	66	7.36	332	−3	15	54	12.56
	R	135	3	12	57	7.89	258	6	18	54	8.70
Basal ganglia											
-pallidum	L	46	−18	6	−3	4.98	77	−15	3	0	12.18
	R	35	15	0	−3	5.12	54	15	3	0	9.10
-caudate	L						81	−12	9	0	7.27
	R						154	15	9	6	7.42
Inferior temporal cortex	L						92	−54	−51	−15	4.98
Superior parietal cortex	L	253	−27	−72	54	8.88	362	−27	−66	45	9.77
	R	155	21	−78	54	6.55	229	39	−66	51	7.48
Inferior parietal cortex	L	431	−30	−63	42	8.97	610	−39	−60	48	10.66
	R	251	51	−45	45	5.99	465	42	−45	42	9.68
Cerebellum	L						842	−9	−78	−34	6.39
	R						1177	35	−63	−39	11.07

L =  left, R  =  right.

**Table 3:** Control>FM activation two sample t-test (*P*<0.05 small volume corrected for multiple comparison and minimum cluster size of 16).

			Coordinates				
	Side	Cluster size	x	y	z	Peak T	p-value
Ventrolateral prefrontal cortex	L	59	−33	21	−21	3.67	0.003
Superior frontal cortex	L	25	−42	18	48	3.14	0.01
	R	26	27	18	57	3.18	0.008
Anterior cingulate cortex	R	27	9	42	15	3.49	0.005
Thalamus	L	49	−6	−24	3	3.67	0.003
	R	6	6	−24	0	2.95	0.01
Middle temporal cortex	R	110	51	−45	18	3.69	0.0001
Inferior parietal cortex	L	24	−42	−69	42	3.42	0.002
	R	41	45	−45	21	5.47	0.001

L =  left, R  =  right.

### 5. Deactivation

Within-group analyses (thresholded at *P*<0.01, false discovery rate (FDR)-corrected for multiple comparisons across the whole brain) of the FM and control groups showed deactivated brain regions in which BOLD activity was less in the 2-back task than in the 0-back task (Fig. 1 and Table 4). The deactivation network included the middle and superior temporal poles, amygdale, hippocampus, parahippocampal gyrus, ventral mPFC, insula, posterior cingulate cortex (PCC), medial parietal cortex, and sensorimotor cortex. Between-group comparisons showed no statistical difference in deactivation network between the two groups.

**Table 4:** 2-back minus 0-back deactivation one sample t-test (FDR corrected for multiple comparison, *P*<0.01 and minimum cluster size of 64).

		Fibromyalgia					Healthy Controls				
	Side	Cluster size	x	y	z	Peak T	Cluster size	x	y	z	Peak T
Sensorimotor cortex	L	218	−17	−39	68	7.34	83	−27	−39	63	3.74
	R	276	18	−39	69	5.28	188	30	−36	63	3.79
Ventral medial prefrontal cortex	L	261	−9	63	2	5.71	375	−6	54	0	9.92
	R	263	6	57	3	5.40	295	3	57	0	6.51
Amygdala	L	18	−24	−3	−24	4.08	43	−24	1	−27	7.90
	R	21	36	0	−24	5.71	49	24	−4	−19	5.63
Hippocampus	L	27	−24	−7	−24	5.10	56	−23	−6	−24	7.20
	R	17	39	−12	−18	5.19	59	24	−3	−21	6.19
Parahippocampal gyrus	L	140	−24	−12	−27	5.88	204	−23	−12	−27	7.11
	R	148	23	−12	−31	5.36	167	27	−9	−30	8.08
Insula	L	237	−36	−14	2	5.32	287	−37	−18	18	8.96
	R	340	36	0	12	6.18	207	39	−12	15	9.79
Posterior cingulate cortex	L	104	−3	−48	21	6.05	118	−6	−48	33	10.91
	R	68	9	−48	21	6.05	49	9	−60	21	8.33
Middle temporal pole	L	59	−33	9	−31	5.23	19	−42	12	−29	4.39
	R						76	54	2	−15	7.90
Superior temporal pole	L	84	−33	9	−27	7.21	16	−42	0	−15	3.51
	R	61	33	9	−27	5.19	85	58	6	−13	6.60
Medial parietal cortex	L	71	−12	−38	72	10.68	66	−6	−26	50	7.11
	R	95	9	−42	69	7.02	83	9	−34	55	6.05

L =  left, R  =  right.

### 6. Correlation between fMRI and Behavioral Data

**Figure 4:**
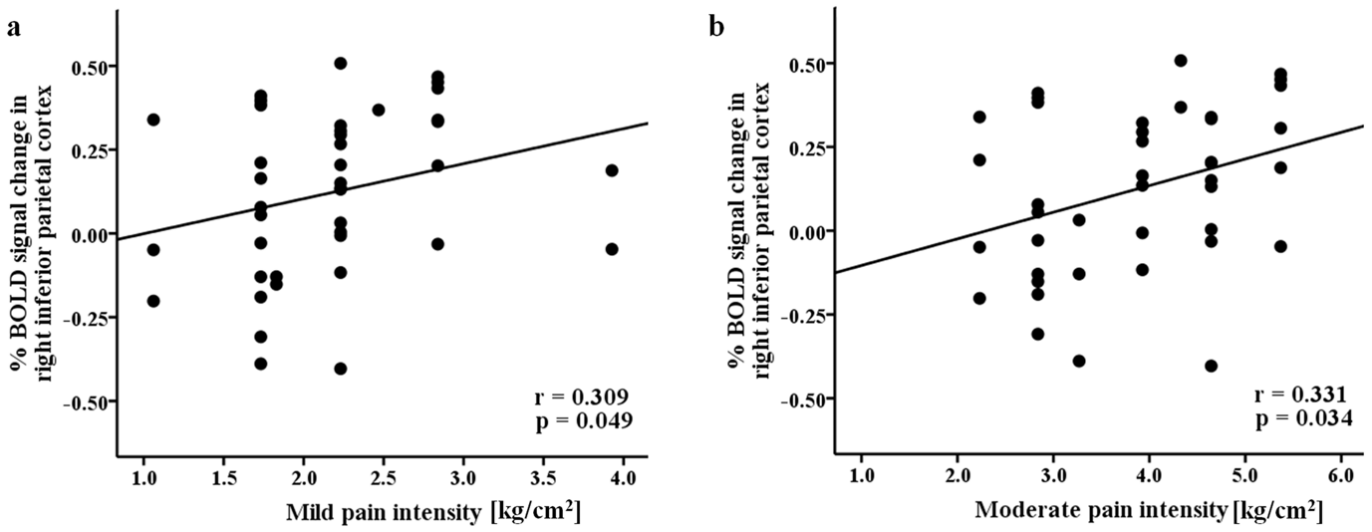
Correlation between BOLD signal change and pressure pain threshold. Percentage BOLD signal change in the inferior parietal cortex showed positive correlation with pressure pain thresholds at (a) mild and (b) moderate pain intensity levels.

Among the brain regions activated during the n-back memory task, the percentage changes of BOLD fMRI signal in the left DLPFC and right VLPFC showed an inverse correlation with BDI (*P*<0.05 and *P*<0.005, respectively) and BAI (*P*<0.05 and *P*<0.005, respectively). In addition, BAI also showed an inverse correlation with the percentage changes of BOLD fMRI signal in the right DLPFC. The association of the left DLPFC and right VLPFC with depression and anxiety was further verified in two-sample between-group analysis. After controlling for both BDI and BAI as covariate factors, the between-group difference in the right VLPFC completely disappeared while the difference partially disappeared in the left DLPFC (Fig. 3b, 3c). Further, after controlling for BAI, the difference disappeared in the right DLPFC (Fig. 3c). The percentage BOLD signal change in the left DLPFC showed a strong positive correlation with 2-back task accuracy (*r* = 0.488, *P* = 0.005) and a negative correlation with 0-back response time (*r* = −0.445, *P* = 0.012). Finally, the percentage changes of BOLD fMRI signal in the inferior parietal cortex, which showed activation even after controlling for both BDI and BAI as covariate factors, showed positive correlation with pressure pain thresholds at mild (*r* = 0.309, *P* = 0.049) and moderate (*r* = 0.331, *P* = 0.034) pain intensity levels (Fig. 4).

## Discussion

The goal of the present study was to elucidate the difference in neural correlates involved in working memory between FM patients and healthy subjects, using fMRI. From a clinical viewpoint, the importance of our fMRI study on working memory in patients with FM is that while pain is the defining characteristic of FM, the disorder is also commonly associated with depression and anxiety. Thus, it is an important clinical question whether memory deficit found in FM patients results from pain itself or from depression associated with pain, or from both. The findings of the current study may provide an important clue regarding the neurobiological mechanism for memory deficit that is found in chronic pain syndrome with depression.

In within-group analyses, activation of the cortical network showed a similar distribution in both the healthy subjects and FM patients, prominently including the lateral premotor cortex, dorsal cingulate cortex, medial premotor cortex, DLPFC, VLPFC, and inferior parietal cortex. These findings are consistent with those of a previous study that reported frontoparietal activations while performing the n-back test [17]. In between-group analyses, however, FM patients showed reduced activation in the DLPFC, VLPFC and inferior parietal cortex. During performance of the n-back test, the prefrontal cortex is thought to be a mediator in monitoring a series of stimuli, adjusting information held in the working memory to incorporate the most recently presented stimulus, while rejecting more temporally distant stimuli [17]. With regard to models of cognitive control, the DLPFC maintains the context to provide task-appropriate response [20]. In addition, the VLPFC is concerned specifically with remembering or retrieving during implementation of an intended act or plan [17]. Therefore, structural and functional abnormalities of the prefrontal cortex might contribute to impairments in the maintenance and manipulation of working memory. These impairments may in turn lead FM patients to organize information inappropriately, thereby resulting in forgetfulness and problems with concentration in daily life. A previous voxel-based morphometric study on FM demonstrated that working memory performance was closely correlated with gray matter volume in the prefrontal cortex [13].

Between-group comparisons, controlling for depression and anxiety level, revealed that within the working memory network, the left DLPFC, right VLPFC, and right inferior parietal cortex were associated with the rating of depression and anxiety severity. Our finding of an association of these brain regions with depression and anxiety is consistent with previous reports on working memory tasks in depression and anxiety [21]–[23]. The DLPFC has primarily been associated with “executive” or “cognitive” functions such as the maintenance and manipulation of items in the working memory, and the VLPFC may be associated with the ability in depression to mediate attempts to modulate emotional, cognitive, and behavioral responses [24], [25]. Recent study also demonstrated that the VLPFC is associated with anxiety activity [26]. With respect to the right DLPFC involvement with anxiety, previous magnetic resonance spectroscopy study reported that the abnormality of the *N*-acetylaspartate/creatine ratio, a measure of neuronal viability, was found in the right DLPFC in anxiety disorder patients versus healthy comparison subjects [27]. Therefore, our results suggest that depression and anxiety partially affects the impairment of working memory function in FM patients. More specifically, the neural correlates affected by depression and anxiety are the brain regions that play a role in correcting behavioral or emotional responses and in the maintenance and manipulation of items in the working memory.

After controlling for depression and anxiety level as a covariate, the activity of inferior parietal cortex was still observed in between-group analysis. That is, our data revealed that within frontoparietal working memory network, inferior parietal cortex showed significantly lower activation in the FM group than in the control group even after controlling for depression and anxiety. Furthermore, the neural activity in the inferior parietal cortex showed close correlation with pain threshold in each subjects. Therefore, the impairment in the inferior parietal cortex of the frontoparietal working memory network was associated not only with depression and anxiety but also with pain itself. Previous studies showed functional impairments in the inferior parietal cortex in FM patients. For example, it has been reported that the inferior parietal cortex showed greater perfusion [11] and higher activation in response to nonpainful stimuli [10] in FM patients. While the dorsal inferior parietal cortex plays an important role in maintaining information temporally and switching attention rapidly, the ventral dorsal inferior parietal cortex is associated with phonological encoding and recording processes [28]. Therefore, our data suggest that the ability to effectively process and attend to phonological information could be compromised in FM patients due to pain through functional deficits in the inferior parietal cortex. With regard to brain deactivation associated with working memory, both healthy subjects and FM patients showed a similarly distributed deactivation cortical network, prominently including the mPFC, ACC, PCC, amygdala, and parahippocampal gyrus. This deactivation network included core brain structures found in default mode network (DMN) and is consistent with previous findings that the DMN is deactivated during performance of a working memory task [29], [30]. In the present study, direct comparison of the deactivation networks showed no statistically significant differences in neural deactivation between FM patients and healthy subjects. It is interesting to compare our findings in FM patients with chronic pain with findings in patients with chronic back pain. In an investigation of patients with chronic low back pain [31], Baliki *et al.* demonstrated that patients and healthy controls showed a similar activation pattern when performing a visual spatial attention task, but patients exhibited significantly less deactivation than healthy subjects in the mPFC. In contrast to patients with back pain, however, working memory impairments in FM patients may be attributed to differences in activation of frontoparietal network rather than deactivation of the negative network. Recently, a resting-state fMRI study showed a greater connectivity between the DMN and the insular cortex in FM patients suggesting that intrinsic neural links between the DMN and insula might be hyperactive in FM patients [32]. One possible reason for the potentially discrepant result with resting-state fMRI study may be the difference in the experimental designs. That is, the DMN is deactivated with respect to n-back memory task in our study whereas resting-state fMRI leads to several different intrinsic neural networks without applying a task. Therefore, it is very cautious that the findings on the DMN from different experimental designs are directly compared.

One of the possible limitations of the current study is medication. Since there was no controlling for antidepressants, medication might be a possible confounder in the results of this study. Although we do not exclude the possibility that working memory alteration in FM might be from medication, previous studies in clinical trials of patients with FM demonstrated that either milnacipran or pregabalin did not cause impairments in objective cognitive measures including working memory [33], [34]. Furthermore, the direct comparisons (i) between patients without medications and patients with medications and (ii) between all patients and patients with medications showed that there were no differences in activation patterns even at the lowered statistical significance (*P*<0.01 uncorrected). Therefore, it seems unlike that the group differences in brain activation resulted from the systematic effect of a specific drug.

In summary, during the n-back memory task, FM patients showed reduced activation in several brain regions which may be associated with impairments in maintenance and manipulation of working memory. More specifically, within the working memory network, the left DLPFC, right VLPFC, and right inferior parietal cortex were associated with the rating of depression and anxiety severity. On the other hand, inferior parietal cortex was also strongly associated with the pain rating. In addition, our data indicate that there were no differences in deactivation network between FM patients and healthy subjects during performance of the n-back test. Taken together, our results indicate that the working memory deficit found in FM patients may be attributable to differences in neural activation of the frontoparietal memory network and may result from both pain itself and depression and anxiety associated with pain.

## Methods

### 1. Subjects

A total of 41 female subjects (19 FM patients and 22 healthy controls) were enrolled in this study. The subjects were age-matched (38.73±7.65 yrs in the FM group vs. 38.27±8.48 yrs in the healthy controls) and all were right-handed. The healthy controls were recruited volunteers, and all were screened for the presence of chronic widespread pain, generalized weakness, sleep disturbance, and specific tender points. At the time of initial diagnosis, all patients met the classification criteria for FM proposed by the American College of Rheumatology in 1990 [35]. FM patients were recruited consecutively from outpatient rheumatic clinics at four university-based hospitals and from one general hospital. Among 19 patients, seven patients took antidepressants. Six patients have taken pregabalin (75 mg) once daily and one patient has taken both pregabalin (75 mg) and milnacipran (25 mg) once daily.

### 2. Ethics Statement

The study protocol was approved by the Institutional Review Board at Kyungpook National University Hospital (No.74005-1703). All participants agreed to participate in our fMRI study and provided written informed consent.

### 3. Assessment of Activity for FM

Demographic, clinical, and psychological data, including age, education, disease duration, and tender point count were obtained from reviews of medical records and an interview with each participant at the time of study enrollment. Tender points were calculated from direct palpation of 18 specific anatomical locations with a force of 4.0 kg/m^2^ [36]. The functional abilities of FMS patients were assessed using the Korean version of the fibromyalgia impact questionnaire (FIQ) [37]. The severity of depression was evaluated using the Beck depression inventory (BDI) [38] and the Beck anxiety inventory (BAI) [39].

### 4. Pain Threshold Measurement

Pain threshold was assessed before fMRI scan. Discrete pressure stimuli of 5 seconds in duration were applied to the left thumbnail with a 1-cm^2^ hard rubber probe attached to a hydraulic piston. A combination of valves and calibrated weights produced controlled, repeatable stimulation that approached a rectangular waveform. Pressure pain sensitivity was evaluated by subjective scaling of suprathreshold sensation using a combined numerical analog descriptor scale of pain intensity and unpleasantness [40]. Subjects were asked to rate the intensity and unpleasantness of pressure pain sensations evoked by an ascending series of stimuli, beginning at 1.0 kg/cm^2^ and ascending in 0.5 kg/cm^2^ step up to tolerance or to a maximum of 6 kg/cm^2^. Following the ascending series, eight stimuli (intensities of 1.0, 1.5, 2.0, 2.5, 3.0, 3.5, 4.0, and 4.5 kg/cm^2^) were delivered twice in random order. We determine the stimulus intensities necessary to elicit mild and moderate pain ratings. The inter-stimulus interval was 30 seconds.

### 5. 2-back Memory Task

The working memory paradigm consisted of the n-back memory task. The n-back task, where n is an integer (usually 1, 2, or 3), requires on-line monitoring, updating, and manipulation of remembered information, and is therefore assumed to place great demands on a number of key processes within working memory [17]. Participants performed a letter n-back task with two conditions: 0-back and 2-back. In the 0-back condition, participants were asked to remember a target letter that was presented at the beginning of each trial block. In the 2-back condition, they were asked to respond when a letter matched one that had been presented two letters before the present letter. We used letters from the Korean alphabet as target cues. Stimuli were displayed using SuperLab (Cedrus Corp., version 4.5, San Pedro, CA). When SuperLab detects the MRI scan trigger, it immediately starts the n-back stimulus task. The stimuli were presented binocularly using a goggle-based system (modified Silent Vision SV-7021 Fiber Optic Visual System, Avotec Inc., Stuart, FL) positioned on top of the head coil. Participants were asked to press a button with their right index finger if a specific target appeared. For example, in the 2-back task, participants determined whether an item was the same as that two trials back. If the item was the same, participants pressed the button under their right index finger. Participants pressed the button under their right middle finger if the item was different to that presented two trials back. To ensure that the participants understood the task demands, they rehearsed outside the scanner prior to the fMRI investigation, practicing a lettered 0- and 2-back memory task that had the same stimulation timing as the subsequent fMRI paradigm. The experiment utilized a blocked design with two epochs for each of the two experimental conditions (4 epochs in total). Each stimulus letter was visible for 500 ms and was followed by a fixation cross that randomly appeared for 2500 or 3500 ms. Ten letters were presented in each epoch of trials, so that each epoch lasted 36 s. The probability of a letter being a target was 31%. The entire functional scanning run took approximately 4 min 48 sec.

### 6. Functional Magnetic Resonance Imaging

Blood oxygenation level dependent (BOLD) contrast was collected for each subject using a 3.0 T GE EXCITE (Milwaukee, WI) scanner equipped with a transmit–receive body coil and a commercial eight-element head coil array. T2*-weighted echo planar imaging was used for fMRI acquisition. The following acquisition parameters were used in the fMRI protocol: echo time (TE)  = 40 ms, repetition time (TR)  = 3000 ms, field of view (FOV)  = 22 cm, acquisition matrix  = 64×64. Using a midsagittal scout image, 31 contiguous axial slices with 4 mm thickness were placed along the anterior–posterior commissure (AC–PC) plane covering the entire brain. The first three acquisitions were discarded because of T1-saturation effects. A 3-dimensional T1-weighted anatomical scan was obtained for structural reference.

### 7. Functional Image Analyses

Image processing and statistical analyses for fMRI data were carried out using MATLAB (The Mathworks Inc., Natick, MA) and SPM5 (SPM; Wellcome Department of Imaging Neuroscience, London, UK; online at http://www.fil.ion.ucl.ac.uk). The functional images were corrected for sequential slice timing, and all images were realigned to the first image to correct for head movement between scans. The realigned images were then mean-adjusted by proportional scaling and spatially normalized into standard stereotactic space to fit a Montreal Neurological Institute template [41] based on the standard coordinate system.

The pre-processed fMRI data were then entered into first-level individual analysis by comparing fMRI activity during the 2-back task with that during the 0-back (2-back >0-back). In second-level within-group analysis, contrast images from the analysis of individual subjects were analyzed by one-sample *t*-tests, thereby generating a random-effects model, allowing inference to the general population. To evaluate the possible confounding effects of depression and anxiety, group analysis was performed with BDI and BAI as a covariate. The SPM{t}s were thresholded at *P*<0.01, false discovery rate (FDR)-corrected for multiple comparisons across the whole brain. Finally, the resulting activation maps were created and displayed by projection onto an anatomically standardized mean T1 image of all subjects to identify the anatomical correlates of the activity. To make direct comparisons of brain activations between the control and FM patient groups during the 2-back memory task, contrast images for the main effects were assessed using a two-sample *t*-test. SPM{t}s were thresholded at *P*<0.05, FDR-corrected for multiple comparisons at the voxel level in the ventrolateral prefrontal cortex, superior frontal cortex, thalamus, middle temporal cortex, and inferior parietal cortex defined using the Wake Forest University (WFU) PickAtlas utility (http://www.fmri.sfubmc.edu/). Though multiple comparisons at the whole brain level are susceptible to false positive errors, this statistical procedure at the voxel level using WFU PickAtlas utility has been provided a good balance between sensitivity and specificity while allowing for a rigorous control of false positive findings in functional imaging data [42]. Further, to evaluate the possible confounding effects of depression and anxiety, group analysis was performed with BDI and BAI as a covariate. We also performed within- and between-group analysis with the opposite contrast (0-back >2-back) to investigate whether the 2-back memory task was associated with differential patterns of deactivation. The SPM{t}s were thresholded at *P*<0.01, FDR corrected for multiple comparisons across the whole brain and the resulting deactivation maps were created and displayed by projection onto an anatomically standardized mean T1 image of all subjects. In addition, to clarify possible medication effect, the between-group analyses were performed by dividing FM patients into two sub-groups (patients without antidepressants (N = 12) and patients with antidepressants (N = 7)) and were also performed between all FM patients (N = 19) and patients with antidepressants (N = 7).

Estimates of percent signal change during the 2-back task were calculated from the activation regions of each participant using the MarsBaR-software (http://marsbar.sourceforge.net) and ROIs defined by the Anatomical Automatic Labeling (AAL) ROI library [43]. The average signal used in this calculation was based on all conditions and identified as the beta value for the mean column of the regression analysis.

### 8. Statistical Analysis

Clinical and neuropsychological data were compared using student’s *t* tests. The difference in BOLD signal change of activated brain regions between the two groups was examined with two-sample *t* tests. Pearson correlation analyses were used to determine the correlations between mean percentage changes in BOLD fMRI signal in the brain regions, which showed higher activity in between group analysis and BDI, BAI, and pain threshold in individual subjects. We assessed the effects of percent signal change by multiple regression analysis. All statistical analyses were performed using SPSS (v14) software. Statistical significance was defined at *P*<0.05.
